# Genome Scale Metabolic Model of the versatile methanotroph *Methylocella silvestris*

**DOI:** 10.1186/s12934-020-01395-0

**Published:** 2020-07-16

**Authors:** Sergio Bordel, Andrew T. Crombie, Raúl Muñoz, J. Colin Murrell

**Affiliations:** 1 Department of Chemical Engineering and Environmental Technology, School of Insdustrial Engineering, University of Valladolid, Dr. Mergelina s/n, 47011 Valladolid, Spain; 2 Institute of Sustainable Processes, Dr. Mergelina s/n, 47011 Valladolid, Spain; 3 School of Biological Sciences, University of East Anglia, Norwich Research Park, Norwich, NR4 7TJ UK; 4 School of Environmental Sciences, University of East Anglia, Norwich Research Park, Norwich, NR4 7TJ UK

## Abstract

**Background:**

*Methylocella silvestris* is a facultative aerobic methanotrophic bacterium which uses not only methane, but also other alkanes such as ethane and propane, as carbon and energy sources. Its high metabolic versatility, together with the availability of tools for its genetic engineering, make it a very promising platform for metabolic engineering and industrial biotechnology using natural gas as substrate.

**Results:**

The first Genome Scale Metabolic Model for *M. silvestris* is presented. The model has been used to predict the ability of *M. silvestris* to grow on 12 different substrates, the growth phenotype of two deletion mutants (ΔICL and ΔMS), and biomass yield on methane and ethanol. The model, together with phenotypic characterization of the deletion mutants, revealed that *M. silvestris* uses the glyoxylate shuttle for the assimilation of C1 and C2 substrates, which is unique in contrast to published reports of other methanotrophs. Two alternative pathways for propane metabolism have been identified and validated experimentally using enzyme activity tests and constructing a deletion mutant (Δ1641), which enabled the identification of acetol as one of the intermediates of propane assimilation via 2-propanol. The model was also used to integrate proteomic data and to identify key enzymes responsible for the adaptation of *M. silvestris* to different substrates.

**Conclusions:**

The model has been used to elucidate key metabolic features of *M. silvestris*, such as its use of the glyoxylate shuttle for the assimilation of one and two carbon compounds and the existence of two parallel metabolic pathways for propane assimilation. This model, together with the fact that tools for its genetic engineering already exist, paves the way for the use of *M. silvestris* as a platform for metabolic engineering and industrial exploitation of methanotrophs.

## Background

Methane is currently being released into the atmosphere at a rate over 70 million tonnes per year [1]. Given its high green-house potential, which, in a 20 year horizon, has been estimated to be 80 times that of CO_2_ [2], methane emissions constitute a serious environmental concern. Most of the methane emitted into the atmosphere is of biological origin, produced by methanogens in environments such as wetlands, although a substantial amount, 30% [3], occurs as natural gas arising from both anthropogenic and natural sources. This natural gas typically contains other short chain alkanes, principally ethane and propane, in addition to methane. The use of technologies such as fracking is expected to increase the importance of operational releases of natural gas [4]. The localized nature of many of these anthropogenic emissions, makes it possible to implement end-of-pipe abatement technologies. In this context, the use of methanotrophic organisms constitutes an efficient solution for methane abatement, and also offers the possibility of transforming a waste gas into marketable chemicals and commodities [5], thus contributing to the development of a circular economy. The cost of glucose, which is the most commonly used substrate in industrial biotechnology, can account for 30% of the total production costs, which makes methane a very attractive alternative [6].

The use of methanotrophs as platforms for metabolic engineering, requires both the development of techniques for their genetic manipulation and Genome Scale Metabolic Models (GSMMs) to design metabolic engineering strategies. Here we present the first GSMM of *Methylocella silvestris*. This is a versatile methanotroph that grows not only on methane but also on organic acids, alcohols [7] and short chain alkanes such as ethane and propane [8]. Bacteria of the genus *Methylocella* are abundant in many environments, including natural gas seeps [9, 10] and were also found among the microbial community following the Deepwater Horizon incident [11]. This may be linked to their ability to use ethane and propane as carbon sources and suggests that *Methylocella* could be a suitable platform for using natural gas as carbon source in industrial biotechnology, especially as genetic engineering tools have already been developed for this microorganism [12].

Currently, GSMMs have been published for 2 type I (gamma proteobacteria) methanotrophs, namely *Methylomicrobium buryatense* [13] and *Methylomicrobium alcaliphilum* [14], and 4 type II (alpha proteobacteria) methanotrophs of the genus *Methylocystis* [15, 16]. *Methylocella silvestris* is a type II methanotroph that uses the serine cycle for carbon assimilation, but differs from *Methylocystis* species in having a soluble methane monooxygenase (sMMO) [17] and a propane monooxygenase (PrMO) [8], while the *Methylocystis* species have only a membrane-associated particulate methane monooxygenase (pMMO) [18]. The metabolic implications of these differences are discussed later.

Previous work [8] showed that *Methylocella silvestris* oxidizes propane to a mixture of 1-propanol and 2-propanol. Most of the 2-propanol is produced by the soluble methane monooxygenase (sMMO), encoded by the genes Msil_1262–1267, while 1-propanol is mostly produced by a propane monooxygenase (PrMO), encoded by the genes Msil_1651–1648. This was proved by deleting the genes Msil_1262 and Msil_1651, encoding the alpha sub-units of each monooxygenase [8]. Other methanotrophs, such as *Methylococcus capsulatus*, have sMMO enzymes that can oxidize a broad range of alkanes and alkenes [19], however this organism lacks metabolic pathways to assimilate the resulting oxygenated intermediates. *Methylocella silvestris* is able to metabolize the oxidation products of ethane (ethanol and acetate) and, more interestingly, is able to metabolize both 1-propanol and 2-propanol via 2 different pathways which until now have not been fully elucidated.

The GSMM presented here enables the investigation of the distinct features of the metabolism of *Methylocella* in a systematic way, and the exploration of its potential as a platform for metabolic engineering. The model has been validated by testing its ability to predict growth (or absence of growth) on 12 different carbon sources, by comparing predicted and experimental biomass yields on methane and ethanol as well as by predicting the outcome of 2 different gene deletions. We also show how the model can be used as a scaffold for the analysis of proteomic data and serves to identify the enzymes whose change in abundance regulates the shift between different metabolic states and adaptation to different carbon sources.

## Results and discussion

### Metabolic model of *Methylocella silvestris*

A GSMM of *Methylocella silvestris *BL2 was constructed by annotating its genome [Joint Genome Institute (JGI) DSM 15510] using RAST [20] and obtaining an initial draft model with SEED [21]. The draft model was curated as described in Materials and methods. Specific pathways such as those involved in the degradation of propane, have been introduced based both in the genomic annotation and experimental evidence from enzymatic tests and mutants generated by gene knockouts (as described in subsequent sections). The reconstructed model included 681 metabolic genes, 1436 reactions and 1474 metabolites. The model has been made publicly available in SBML format at https://github.com/SergioBordel/ModelsMethanotrophs.

**Fig. 1:**
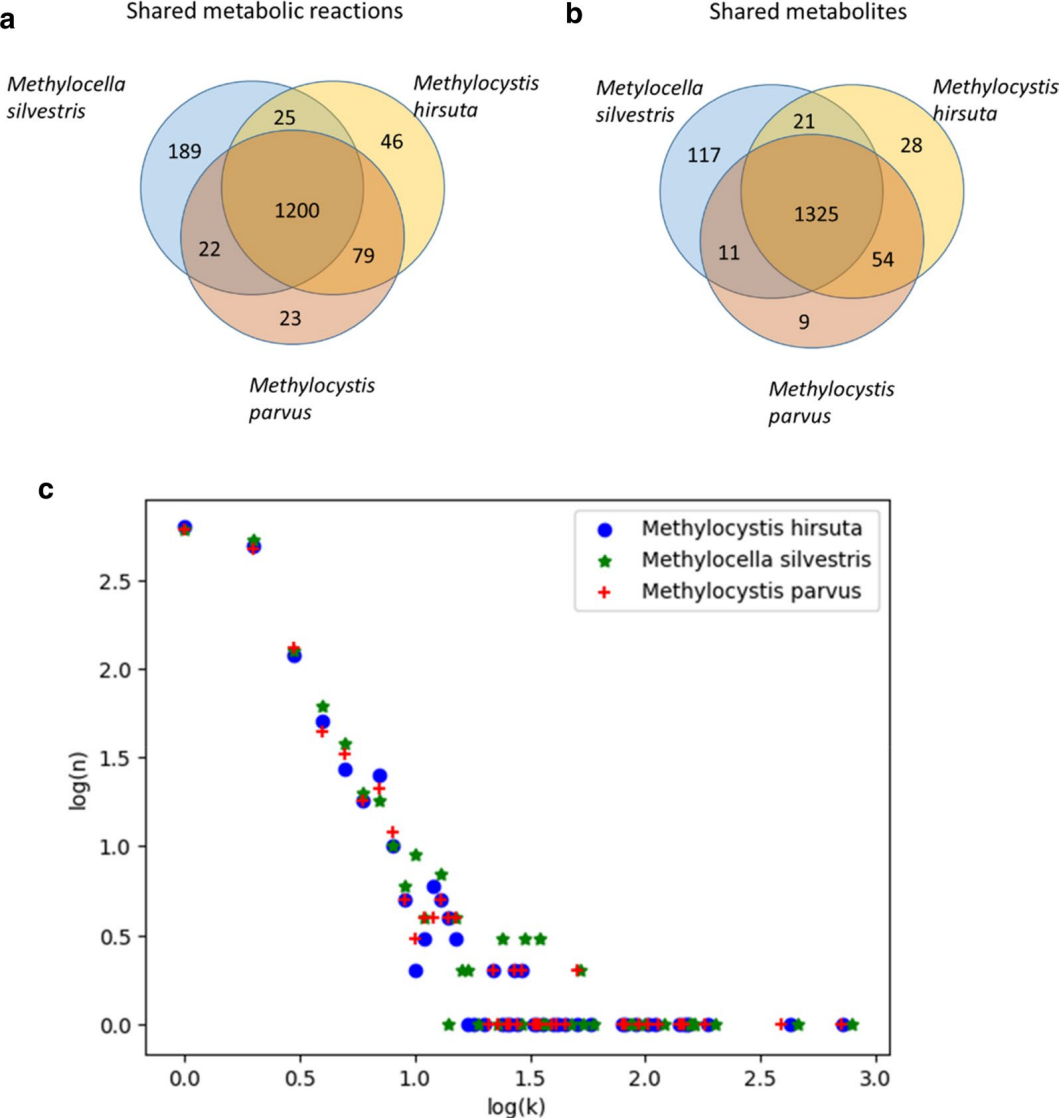
Venn diagrams showing the overlap between the reactions (**a**) and metabolites (**b**) present in the reconstructed GSMMs of *Methylocella silvestris*, *Methylocystis parvus* and *Methylocystis hirsuta*. The plot (**c**) shows the logarithm of the metabolite connectivity (k, the number of reactions in which a metabolite takes part) versus the logarithm of the number (n) of metabolites with connectivity k. The plot shows a typical scale-free topology with a negative slope of 2.5 and an overrepresentation of highly connected metabolites such as ATP and redox co-factors

Figure 1 represents the overlap between the reactions and metabolites present in the *Methylocella* model and those in 2 previously reconstructed models of type II methanotrophs (*Methylocystis hirsuta* and *Methylocystis parvus*) [15, 16], as well as the typical scale-free topology of the metabolic network.

### Differences between the predicted metabolic networks of *Methylocella* and *Methylocystis species*

A complete list of reactions and metabolites specific to *Methylocella silvestris* is provided as Additional file 1: SF1. Among its specific reactions, the metabolic network of *Methylocella silvestris* contains those catalyzed by the enzymes isocitrate lyase (EC 4.1.3.1; Msil_3157) and malate synthase (EC 2.3.3.9; Msil_1325), which form the glyoxylate shunt, allowing the use of ethanol and acetate as sole carbon sources. Other type II methanotrophs such as *Methylocystis* sp. SB2 and *Methylocystis hirsuta* [15, 22] can grow on acetate and/or ethanol, however they do not utilize the glyoxylate shunt but rely on the ethylmalonyl pathway (which is absent in *Methylocella silvestris*) and have very low specific growth rates (0.08 ± 0.006 day^−1^ for acetate grown *M. hirsuta* versus 0.05 ± 0.001 h^−1^ for *Methylocella silvestris* grown on acetate [12]). The role of isocitrate lyase in the growth of *Methylocella silvestris* using ethane and acetate, and also growth on C1 compounds, will be discussed in detail in the following section.

Among the metabolites specific to *Methylocella silvestris* and absent in both of the *Methylocystis* species considered, we predicted 6-phosphogluconate and its derivatives 6-phosphogluconate-1–5-lactone and 2-keto-3-deoxy-6-phosphogluconate, which are involved in the Entner-Doutdoroff pathway. Other specific metabolites are 4-hydroxy-2-oxovalerate and 2-keto-4-pentenoate, which are linked to the presence of a 2-keto-4-pentenoate hydratase (EC 4.2.1.80; Msil_1477).

### Metabolic roles for isocitrate lyase (ICL) and malate synthase (MS)

*Methylocella*, compared to other methanotrophs, is characterized by its metabolic versatility and the high variety of substrates on which it can grow. In particular, its use of alkanes such as ethane and propane [8] is of great interest. The ability to grow on ethane and its metabolic intermediates ethanol and acetate can be explained by the presence of ICL and MS.

In order to confirm the activity of these enzymes, enzyme assays were carried out with extracts of acetate grown *Methylocella silvestris* (see Materials and methods). The measured activities were 23.2 ± 3.3 and 424 ± 59 nmol min^−1^ mg-protein^−1^ for ICL and MS respectively. The metabolic model was used to simulate growth on acetate by setting the specific growth rate equal to its experimental value of 0.05 h^−1^ and minimizing the acetate uptake rate (which is equivalent to a yield optimization). The predicted metabolic fluxes are 0.78 and 0.71 mmol h^−1^ g-DW^−1^ for ICL and MS. Enzymatic activities are reported in nmol min^−1^ mg-Protein^−1^. Thus, to compare the predicted metabolic fluxes with the results of enzyme activity tests, we need to transform grams of dry weight into milligrams of protein. Assuming that half of the total biomass corresponds to proteins, the predicted in-vivo activities of these enzymes are equivalent to 26 and 24 nmol min^−1^ mg-Protein^−1^. The predicted activity of ICL is close to the measured activity (26 versus 23 nmol min^−1^ mg-Protein^−1^), whilst the measured activity of MS is one order higher than the minimal activity necessary to support the observed growth rate (424 versus 24 nmol min^−1^ mg-Protein^−1^).

**Fig. 2:**
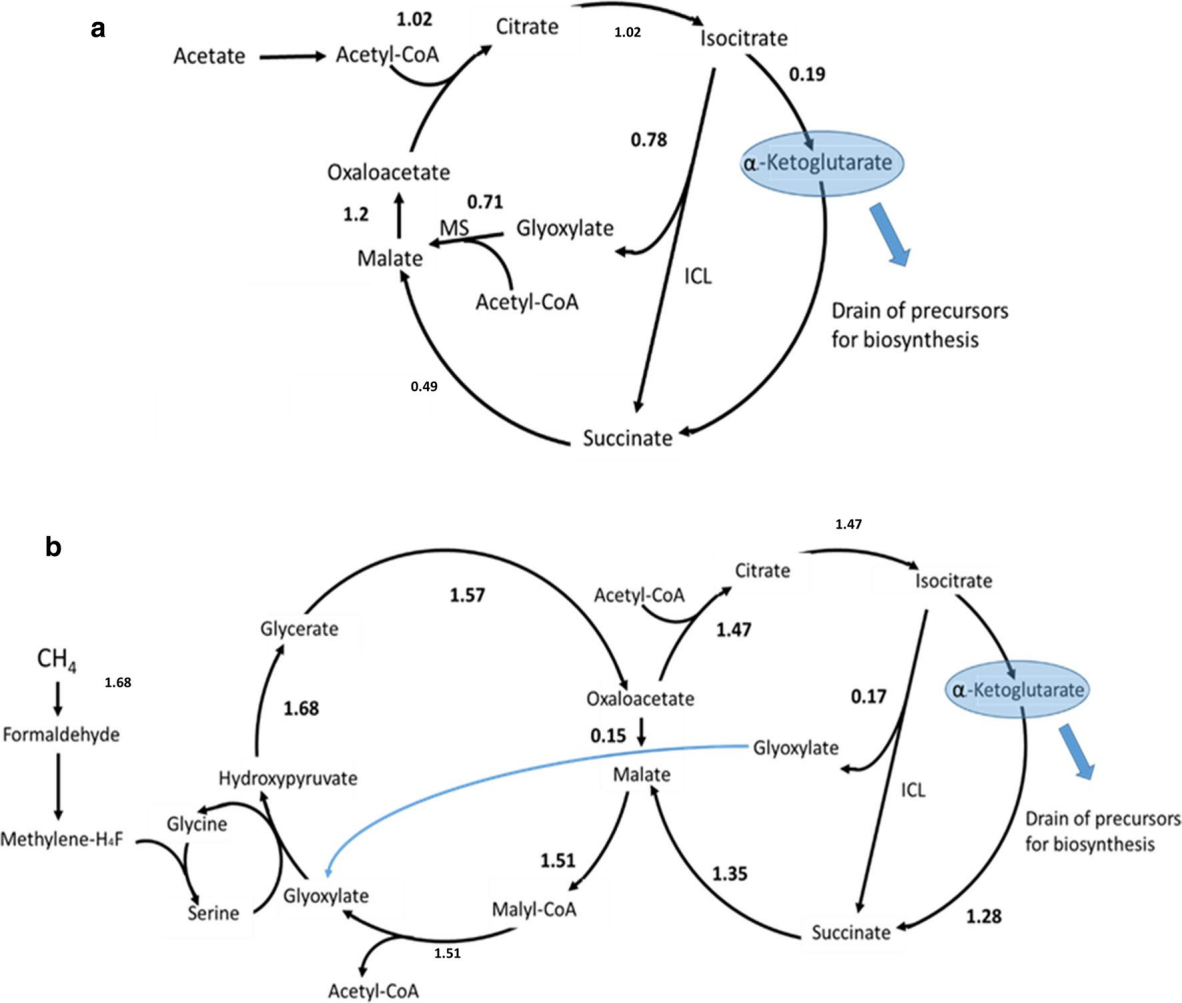
Metabolic flux distributions (fluxes in mmol h^−1^ g-DW^−1^) predicted by the model for growth on acetate (**a**) and methane (**b**). As shown in (**b**), MS activity is not required for growth on C1 substrates (methane, methanol and methylamine), which is consistent with the ability of ΔMS to grow on these substrates. The figure shows only the main metabolic fluxes relevant for our discussion. The depicted metabolites are also consumed and produced by other metabolic reactions

**Fig. 3:**
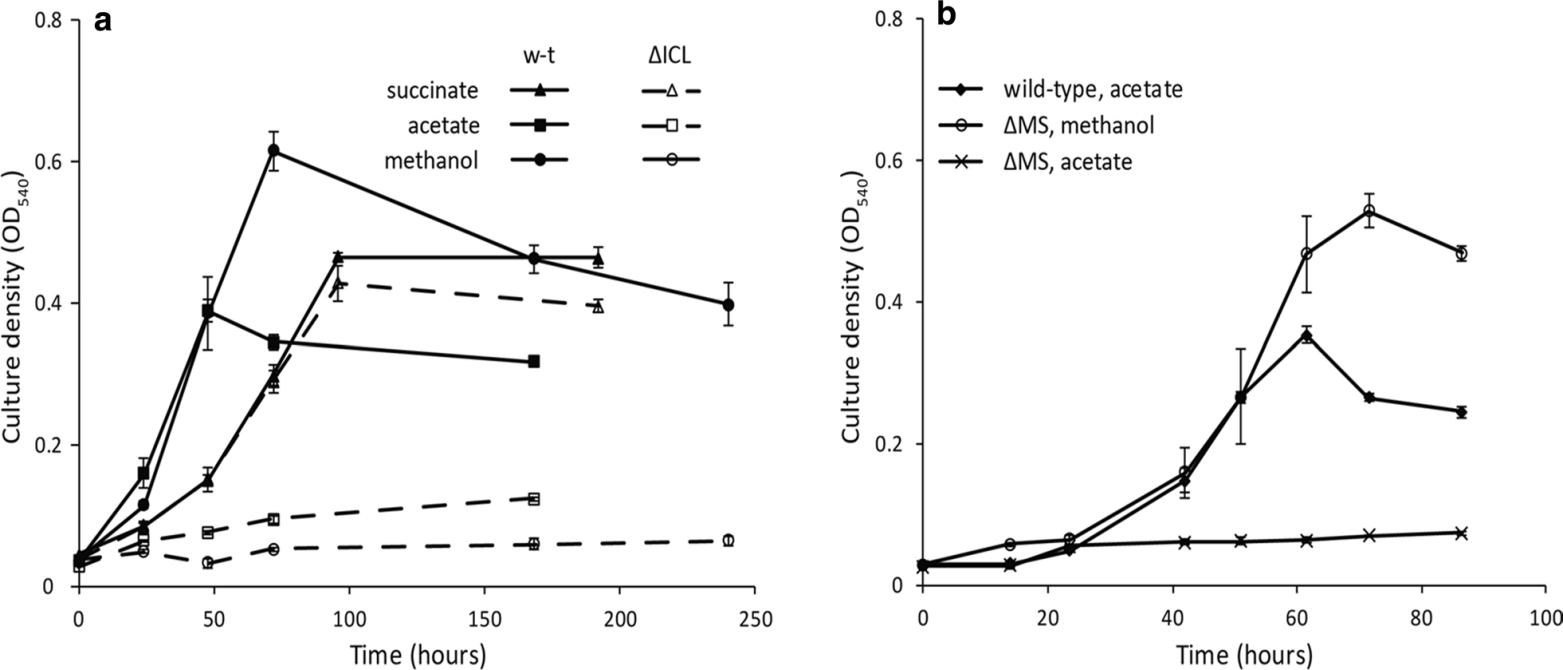
**a** Growth curves of the wild type and ΔICL on succinate; acetate and methanol, ΔICL stops growing on acetate and methanol while keeps its ability to grow on succinate. **b** Growth curves of the mutant ΔMS; only growth on acetate is affected by the deletion of MS

Deletion mutants ΔICL and ΔMS for *M. silvestris* were constructed (see Materials and methods). As expected, both mutants lost the ability to grow on acetate and ethanol. Interestingly, the ΔICL strain also lost its ability to grow on C1 substrates, while the strain ΔMS retained this ability. This suggests that ICL is also playing an anaplerotic role in the serine cycle for the assimilation of C1 compounds. In previous metabolic models of *Methylocystis* strains [15, 16] this anaplerotic role was filled by a glycine synthase that transforms methylene-tetrahydrofolate into glycine (even though this has not been confirmed experimentally). The phenotypic observations for ΔICL and ΔMS suggest that in *Methylocella silvestris*, ICL performs this anaplerotic role by supplying the glyoxylate produced to the serine cycle (Figs. 2). Figure 3 shows the effects of deleting ICL and MS respectively.

**Fig. 4:**
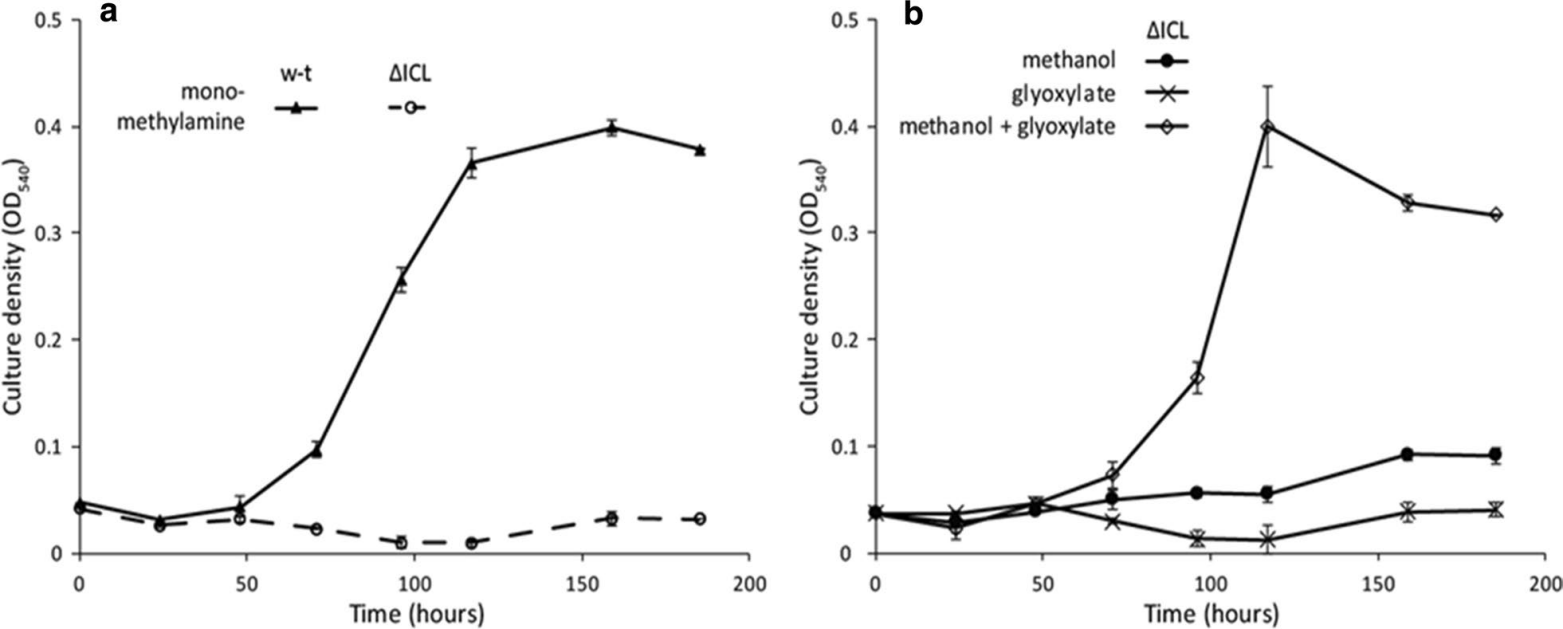
**a** Growth curves of the wild type and ΔICL on methylamine; ΔICL lost its ability to grow on C1 compounds. **b** The ability of ΔICL to grow on C1 compounds is rescued by adding glyoxylate to the medium, however *Methylocella silvestris* is not able to grow on glyoxylate

The role of ICL in supplying glyoxylate to the serine cycle was further confirmed by the fact that addition of glyoxylate to the growth medium rescued the ability of the cells to grow on C1 compounds (Fig. 4b).

### Qualitative model predictions. Growth on different substrates of the wild type and deletion mutants

**Fig. 5:**
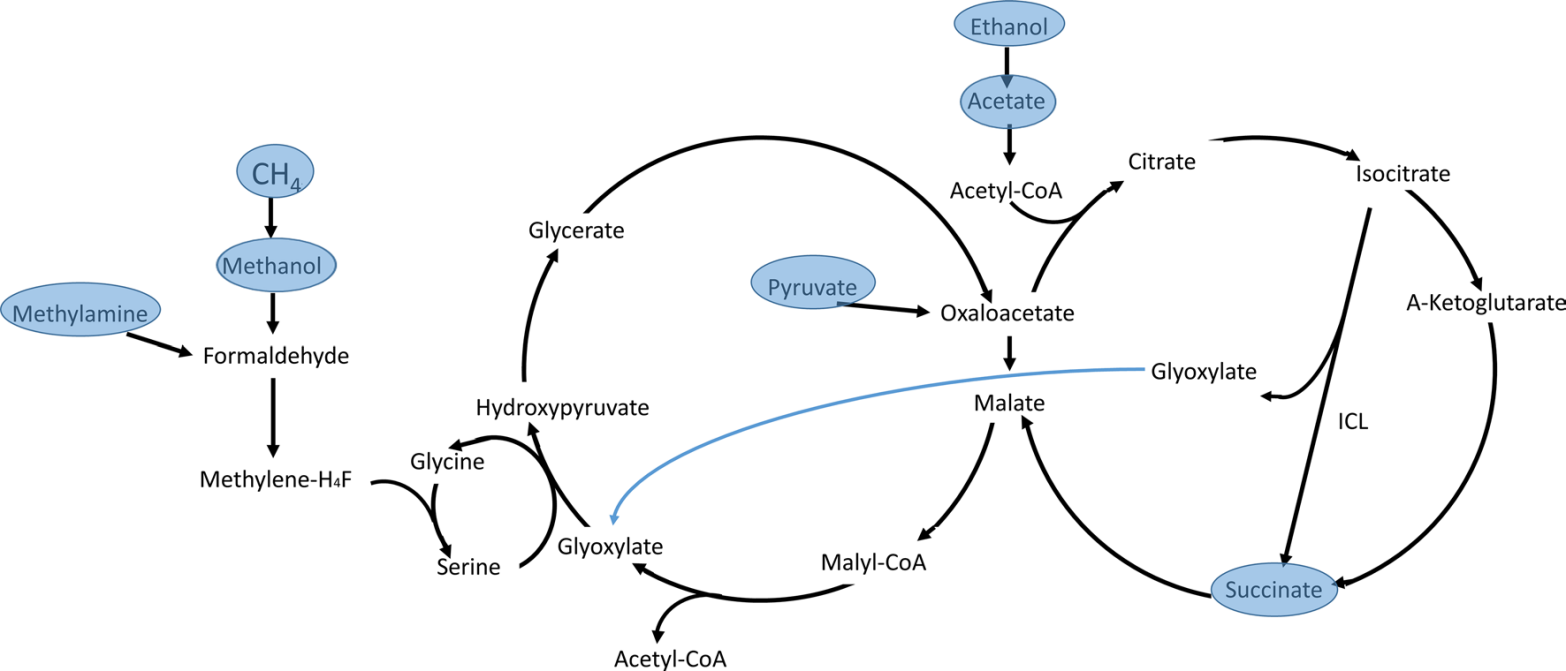
Schematic representation of the central carbon metabolism of *Methylocella silvestris* and the carbon sources in which it grows

*Methylocella silvestris* has been shown to grow on a broad scope of substrates [8], while it cannot grow on substrates such as glyoxylate, oxalate, glycine or urea. Figure 5 summarizes the different substrates on which the wild type grows.

Table 1 represents the ability of the wild type and the mutants ΔICL and ΔMS to grow on 12 different substrates predicted by the model. The model predictions match the experimental observations in all the cases.

**Table 1:** Growth on different carbon sources

	Wild type	ΔICL	ΔMS
Methane	** + **	**−**	** + **
Methanol	** + **	**−**	** + **
Methylamine	** + **	**−**	** + **
Propane	** + **	** + **	** + **
Ethanol	** + **	**−**	**−**
Acetate	** + **	**−**	**−**
Pyruvate	** + **	** + **	** + **
Succinate	** + **	** + **	** + **
Glyoxylate	**−**	**−**	**−**
Oxalate	**−**	**−**	**−**
Glycine	**−**	**−**	**−**
Urea	**−**	**−**	**−**

Experimental observations and model predictions are coincident for all the metabolites included in the table

### Quantitative predictions

**Fig. 6:**
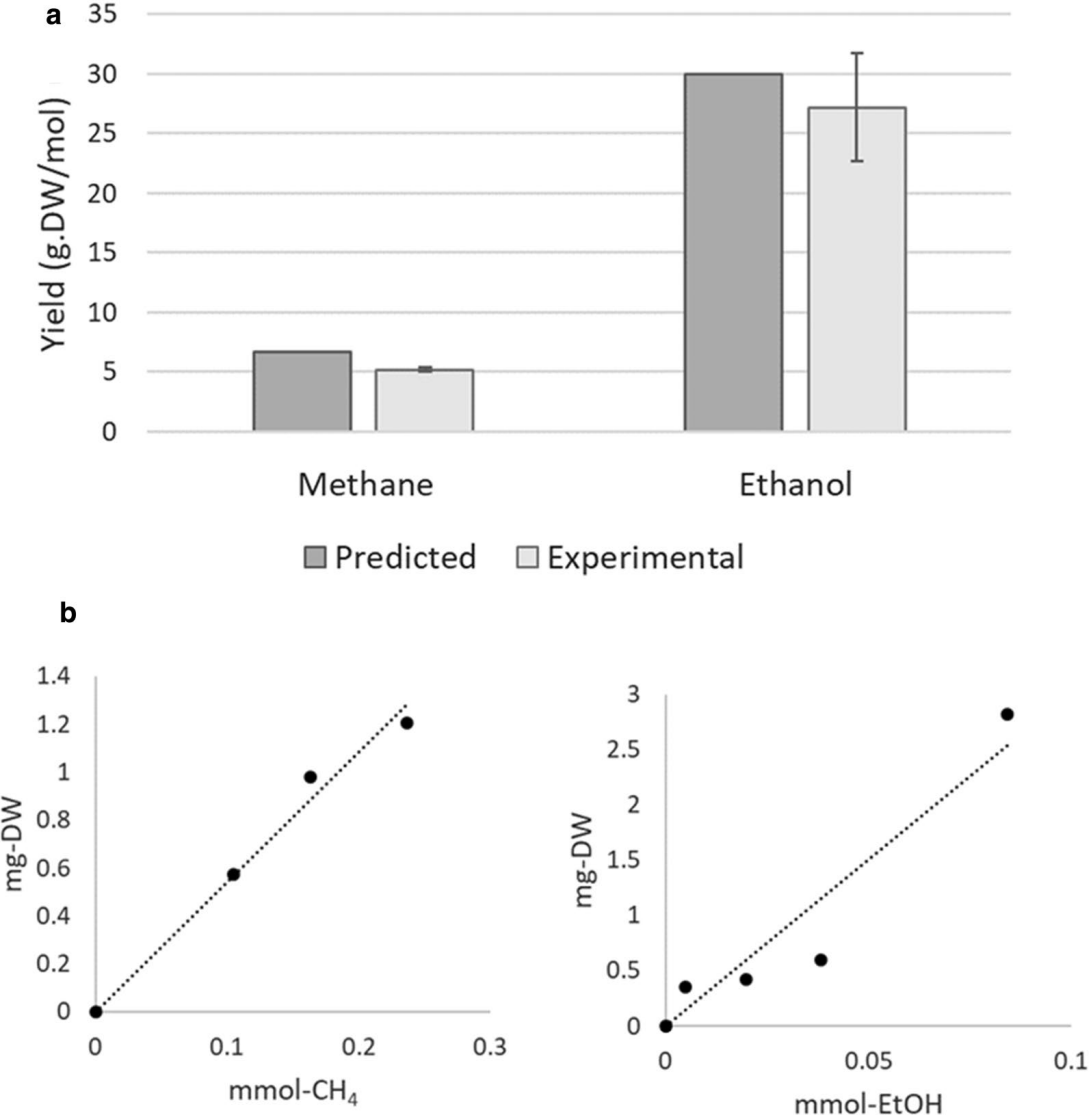
**a** Theoretical maximal yields versus measured yields on methane and ethanol; error bars are standard deviations (n = 3). **b**Biomass produced versus consumed substrate for one of the replicates

Biomass yields on methane and ethanol (Fig. 6) were obtained by monitoring over time biomass growth and substrate consumption, and calculating the slope of biomass produced versus substrate consumed (see Materials and methods). Maximal theoretical yields were obtained by constraining the substrate uptake rate and maximizing the production of biomass. No ATP maintenance consumption was introduced in the simulations; therefore, the results represent the maximal theoretical value. The difference between the theoretical and the experimental values can be explained by ATP maintenance requirements of 3.45 mmol h^−1^ g-DW^−1^. The estimated value for *Methylocystis hirsuta* was 1.25 mmol h^−1^ g-DW^−1^ [15].

The yield of *Methylocella silvestris* on methane is lower, both theoretically and experimentally, than those of *Methylocystis* species [15]. Experimental yields on methane were 5.2 ± 0.17 g-DW mol^−1^ for *Methylocella silvestris* versus 7.7 ± 0.26 g-DW mol^−1^ for *Methylocystis hirsuta*. The metabolic model predictions are 6.64 and 8 g-DW mol^−1^ respectively. The difference between both strains is in part related to the redox cofactors used by sMMO and pMMO, with sMMO using NADH, while the pMMO, characteristic of *Methylocystis*, uses ubiquinone [15], which itself is reduced in complex I of the respiratory chain coupled to proton extrusion, contributing in this way to higher ATP production.

### Propane degradation pathways

Two reactions have been incorporated into the model, with respective identifiers “PrMO” (yielding 1-propanol) and “PrMO2” (yielding 2-propanol). Even if some cross activity is possible, the reaction “PrMO” has been annotated as catalyzed by the enzyme PrMO, while the reaction “PrMO2” has been annotated as catalyzed by the enzyme sMMO. Both monooxygenation reactions are coupled to the consumption of an oxygen molecule and the oxidation of an NADH equivalent. *M. silvestris* grows on both 1-propanol and 2-propanol, although 1-propanol appears to have toxic effects and growth on this substrate is observed only for inocula pre-cultured with propane, but not with other substrates [8]. Growth on 2-propanol was observed in all the cases, independently of the substrate on which the cells were precultured.

The genome of *M. silvestris* contains eight genes predicted to encode NAD(P)-dependent alcohol dehydrogenases (ADHs), as well as a pyrroloquinoline quinone (PQQ)-containing methanol dehydrogenase (Msil_0471) and a PQQ-containing ADH with 73% identity to *xoxF* from *Methylobacterium extorquens* (Msil_1587). For modelling purposes, it is essential to elucidate if the oxidation of 1-propanol and 2-propanol, to propanal and acetone respectively, is coupled to NAD(P)H generation, or coupled to transfer of electrons to PQQ, which are further transferred to cytochrome-c, with proton extrusion [14]. Oxidation by PQQ-containing dehydrogenases was confirmed by assaying the rate of dichlorophenolindophenol reduction in extracts of propane grown cells (using spectrophotometric measurements). Both 1-propanol and 2-propanol were oxidized at similar rates, while NAD and NADP coupled oxidation rates were negligible in both substrates (Table2).

**Table 2:** Quinoprotein, NAD and NADP-dependent activities (± standard deviations with triplicate measurements) in cell extracts, measured in nmol min^−1^ mg-protein^−1^

	1-propanol	2-propanol
PQQ	363 ± 7 nmol min^−1^ mg-protein^−1^	381 ± 30 nmol min^−1^ mg-protein^−1^
NAD	0.6 ± 1 nmol min^−1^ mg-protein^−1^	1.5 ± 1 nmol min^−1^ mg-protein^−1^
NADP	3.3 ± 2 nmol min^−1^ mg-protein^−1^	0 nmol min^−1^ mg-protein^−1^

**Fig. 7:**
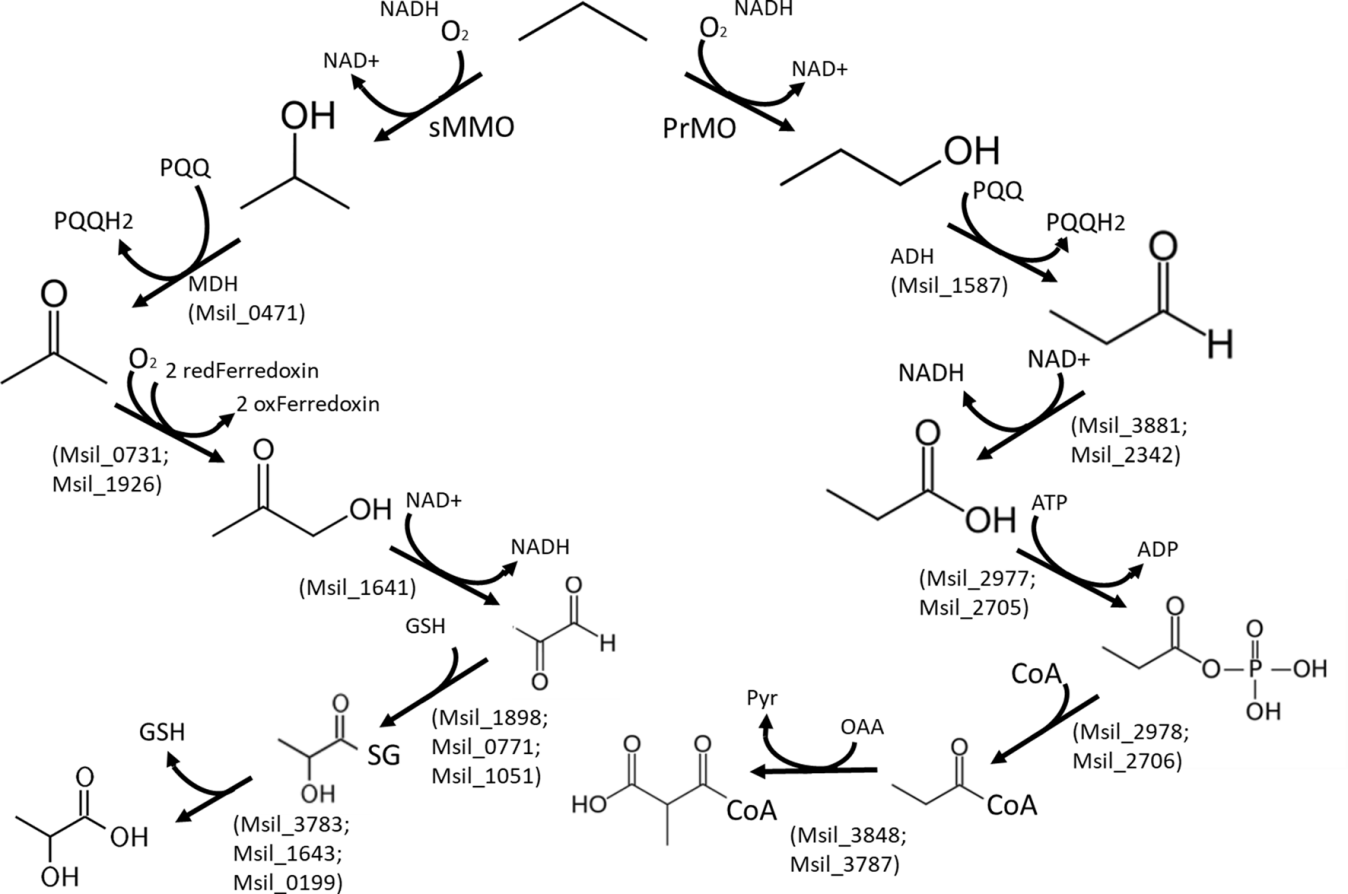
Two alternative metabolic pathways for propane oxidation

*Methylocella silvestris* can grow on both acetone and propanal (the oxidation products of 2-propanol and 1-propanol). The genome revealed the presence of genes encoding a putative pathway from propanal to methylmalonyl-CoA, composed of four reactions with two putative enzymes for each of them (Fig. 7). The existence of this pathway was further supported by the higher activity of methylmalonyl-CoA metabolism in propane-grown cells of *Methylocella silvestris* revealed by proteomics [23].

**Fig. 8:**
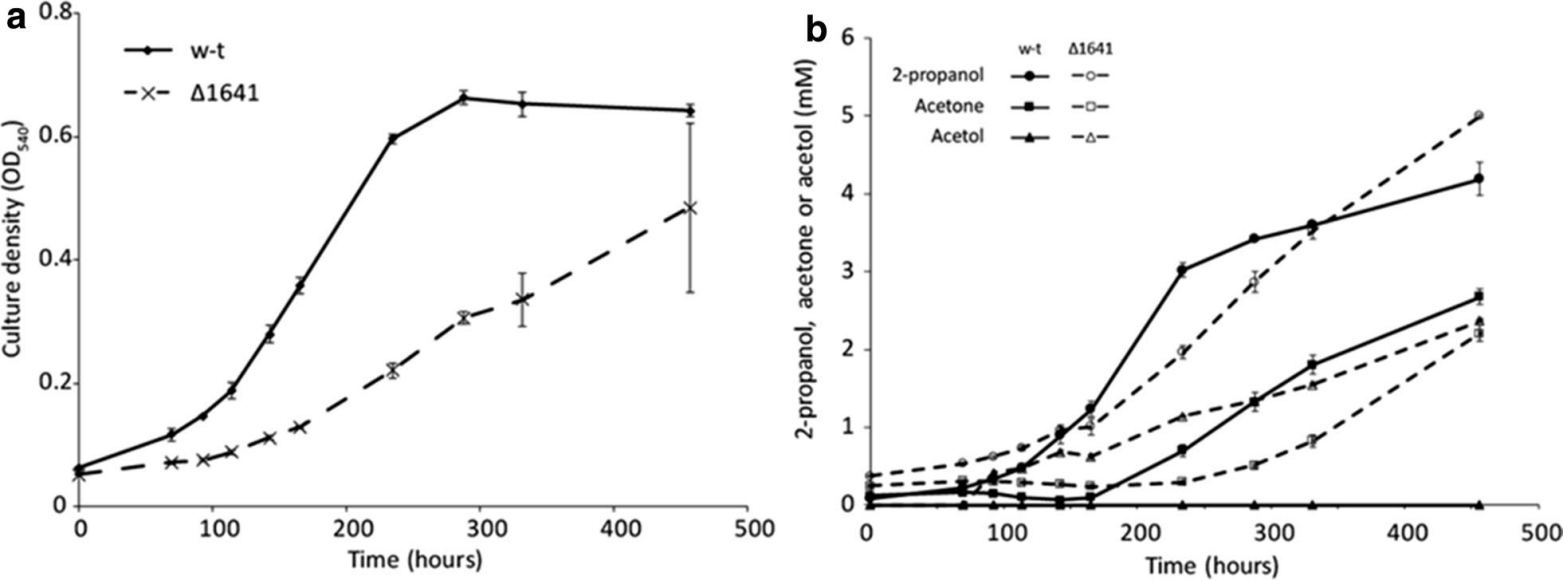
Growth curves on propane of the wild type and the Δ1641 mutant (**a**). Accumulation of 2-propanol, acetone and acetol in the growth medium, for the wild type and the Δ1641 mutant (**b**)

The genome annotation did not reveal all the enzymes catalyzing the reactions involved in the downstream metabolism of acetone. Both 2-propanol and acetone accumulated in the culture broth, during growth on propane, which suggests a kinetic bottleneck downstream of acetone. The RAST annotation shows the presence of three putative hydroxyacylglutathione hydrolases (EC 3.1.2.6; Msil_1898, 0771 and 1051) and three putative lactoylglutathione lyases (EC 4.4.1.5; Msil_3783, 1643 and 0199). These reactions would allow the transformation of 2-oxopropanal into lactate, which could be further metabolized via its transformation into pyruvate by a putative lactate dehydrogenase present in the genome (Msil_2429, 2430, 2431). However, the enzymes involved in the transformation of acetone to 2-oxopropanal remain to be elucidated. Proteomics has revealed strong overexpression of the gene Msil_1641, induced by propane. This gene has been annotated as a glucose-methanol-choline oxidoreductase, which does not suggest any specific association with propane degradation, therefore its role in *M. silvestris* was tested experimentally by constructing a Δ1641 mutant. The deletion mutant showed a lower specific growth rate on propane (0.0075 ± 0.0003 h^−1^ versus 0.0174 ± 0.0002 h^−1^ for the wild type), which confirmed its relation to propane utilization. More interestingly, when growing on propane, the Δ1641 strain started accumulating acetol in its growth medium (besides 2-propanol and acetone, which also accumulated in the wild type strain). The mutant strain also lost its ability to grow on 2-propanol and acetone, which confirms the involvement of Msil_1641 in the downstream degradation pathway of acetone. Based on the evidence alone (and its annotation by RAST as a NAD associated oxidoreductase), it was assumed that Msil_1641 catalyzes the oxidation of acetol to 2-oxopropanal with concomitant NADH production. The oxidation of acetone to acetol was proposed to be catalyzed by two putative cytochrome P450 oxidoreductases represented in the genome (Msil_0731 and Msil_1926). Similar enzymes have shown to catalyze this reaction in rabbit [24]. This reaction is coupled to oxygen consumption and oxidation of ferredoxin as it is shown in Fig. 8.

### Quantitative aspects of propane utilization

There are two alternative pathways for propane degradation in *M. silvestris*, one of them, via 1-propanol, transforms propane into methylmalonyl-CoA, while a second pathway, via 2-propanol, transforms propane into lactate. Both pathways co-exist simultaneously and the metabolic flux going through each of them depends on the ratio between 1-propanol and 2-propanol produced by PrMO and sMMO [8]. Deletion of the gene Msil_1641 truncated the second pathway and led to the accumulation of 2-propanol, acetone and acetol. Batch experiments, in which Δ1641 was grown in serum flasks with 20% propane in the headspace, revealed a specific growth rate of 0.0075 ± 0.0003 h^−1^ and specific production rates of 2-propanol, acetone and acetol of respectively: 0.188, 0.075 and 0.152 mmol h^−1^ g-DW^−1^. This rate of 0.152 mmol h^−1^ g-DW^−1^ was assumed to be equal to the rate of acetol oxidation to 2-oxopropanal in the wild type.

The wild type, which showed a growth rate on propane of 0.0174 ± 0.0002 h^−1^, produced 2-propanol and acetone at 0.189 and 0.073 mmol h^−1^ g-DW^−1^ respectively (with no acetol accumulation in the medium). These experimental values, as well as the acetol oxidation rate of 0.153 mmol h^−1^ g-DW^−1^, inferred from the behaviour of Δ1641, were imposed as constraints in the metabolic model. Propane uptake rate was minimized, leading to a prediction of 0.67 mmol h^−1^ g-DW^−1^. This corresponds to a biomass yield on propane equal to 25 g-DW mol^−1^, which is very close to the experimental yield measured for the wild type of 24 ± 2 g-DW mol^−1^ [8]. This yield was reduced to around one third in the ΔPrMO mutant [8], which is consistent with a higher fraction of propane being transformed to 2-propanol and metabolized through a less efficient metabolic pathway.

### Integration of proteomic data

Proteomic data for *Methylocella silvestris* have been obtained for growth on methane, succinate and propane [23]. The proteome was characterized using reversed phase Ultra-high Pressure Liquid Chromatography (UPLC) prior to mass spectrometric analysis. The numbers of detected proteins (for the most sensitive experimental replicas) were respectively: 672 for cells grown on propane, 802 for cells grown on succinate and 684 for cells grown on methane. Among these proteins, the following numbers of metabolic enzymes (included in the model) were found: 213 for propane, 263 for succinate and 221 for methane.

The reconstructed model was used to calculate correlation coefficients between the predicted metabolic fluxes in each to the three experimental conditions and the enzyme abundances. This allows to identify metabolic reactions whose predicted fluxes correlate with the abundances of the enzymes catalyzing them (or in other words, it aims at finding enzymes with high metabolic control coefficients). Using the terminology of Metabolic Control Analysis (MCA) [25], some enzymes have high metabolic control coefficients, which are defined as the derivatives of the logarithm of the enzyme reaction rate with respect to the logarithm of the enzyme concentration, (a control coefficient of one would mean a linear correlation between the enzyme abundance and the rate of the reaction that it catalyzes) while others have low control coefficients (changes in its abundance have a low impact on the rate of the reactions that they catalyze, a control coefficient of zero meaning an absence of correlation between enzyme abundance and metabolic flux). These control coefficients are properties of the whole metabolic network and cannot be inferred from the kinetics of single enzymes alone. Note that the control coefficient of an enzyme is unrelated to the absolute value of its metabolic flux or to the magnitude of its kinetic constants. It is rather related to the change of metabolic flux caused by changes in its abundance.

These enzymes are responsible for the transcriptional adaptation of the cell to different carbon sources. Model predictions were carried out by setting specific growth rates to their experimental values and minimizing the carbon source uptake rate. In the case of propane, the fluxes in the pathway via lactate (discussed in the previous section), were also constrained to their experimental values. Pearson correlation coefficients and *p* values were obtained for each of the metabolic enzymes detected in the proteome. The p-vales were corrected for multiple testing and a false discovery rate of 0.05 was set as a cutoff. Nine proteins passed this threshold, among them the three subunits of PrMO, which are expressed only in presence of propane. Two other enzymes were expressed only under growth on propane, those were the previously discussed oxidoreductase coded by the gene Msil_1641, and the methylmalonyl-CoA mutase coded by Msil_3785. The identified enzymes included also two enzymes of the serine cycle, namely phosphoenolpyruvate carboxylase (Msil_1718) and serine-glyoxylate aminotransferase (Msil_1714), which show their highest expression levels during growth on methane. 3-oxoacyl-synthase (Msil_2205) and inorganic pyrophosphatase (Msil_1025) showed their highest expression and metabolic flux during growth on succinate, followed by growth on propane and methane.

**Fig. 9:**
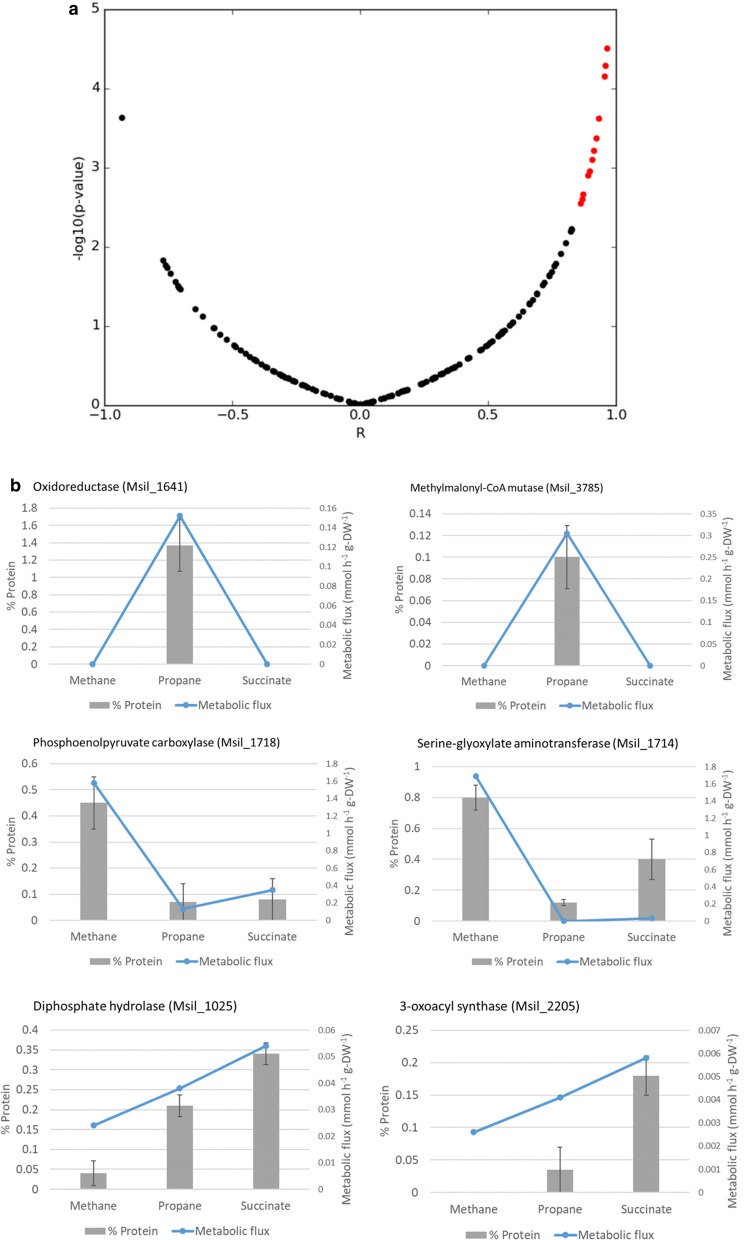
**a** Pearson correlation coefficients and *p* values for each metabolic enzyme, the red points represent those with a false discovery rate below 0.05 after correcting for multiple testing. **b** Protein abundances (left side axis) and predicted metabolic fluxes in mmol h^−1^ g-DW^−1^ (right side axis) for 6 of the significantly correlated enzymes

Figure 9a shows a plot of the Pearson correlation coefficients between protein expression and predicted reaction rates in each of the three conditions, versus minus the logarithm of the *p* value resulting from the correlation. Figure 9b illustrates the protein abundances measured under the three studied conditions and the metabolic fluxes in the corresponding reactions predicted by the model (for 6 of the better correlated enzymes).

## Conclusions

In this work we present the first GSMM of an organism of the genus *Methylocella*. The model captures the metabolic versatility of the strain and is able to reflect its ability to grow on a broad range of substrates as well as to predict quantitatively its biomass yields on methane and ethanol. The model also predicts the phenotypes of two deletion mutants, ΔICL and ΔMS, and could explain the reliance of *M. silvestris* on the glyoxylate shunt for growth on C1 substrates. This feature makes *M. silvestris* different from other type II methanotrophs and should be taken in account for the design of future metabolic engineering strategies. Regarding the ability of *M. silvestris* to grow on propane, two alternative pathways have been identified. Experimental evidence suggests that both pathways operate in parallel. One of the pathways proceeds through methylmalonyl-CoA while the other proceeds through lactate. The existence of 2 independent pathways for propane assimilation makes *M. silvestris* a potential platform to synthesize compounds using propane as a carbon and energy source. For example, the deletion of lactate dehydrogenase can be expected to disrupt the second pathway and to result in lactate accumulation, while energy and biomass could still be obtained via the first pathway. Alternatively, the second pathway could be disrupted downstream of methylmalonyl-CoA and its metabolic flux deviated to compounds having methylmalonyl-CoA as precursor, while the cell could still use the pathway via lactate to obtain energy and biomass precursors.

## Materials and methods

### Reconstruction of the Genome Scale Metabolic Model

The genome of *Methylocella silvestris* BL2 was obtained from the Joint Genome Institute (JGI) (DSM 15510). After annotation with RAST [20] a draft model was generated using SEED [21]. The gene identifiers used in the text correspond to the original annotation, in order to maintain consistence with previous literature, however the model has been constructed based on the RAST annotation provided as Additional file 2: SF2. Additional file 3: SF3 contains the equivalences between the identifiers of both annotations. The draft model was manually curated following the steps previously described [15], which involved reviewing all the genes annotated as having enzymatic activities and checking that they are associated to the correct reactions in the model, correcting the reversibility of reactions in order to avoid thermodynamically unfeasible production of ATP or extrusion of protons, as well as correcting the stoichiometries of some reactions, in particular those involved in the respiratory chain. Some RAST annotations were corrected using genetic evidence, for example the genes Msil_1649, 1650 and 1651 were annotated by RAST as sMMO sub-units, while it had been previously shown experimentally that they encode a propane monooxygenase that cannot oxidize methane [8]. The genetic evidence previously discussed also shows that Msil_1641, which had been annotated by RAST as a glucose-methanol-choline oxidoreductase, is in fact responsible for the oxidation of acetol.

The reconstructed GSMM in SBML format (Methylocella.xml), together with tab separated files containing metabolites (Methylocella_metabolites.txt) and reactions (Methylocella_reactions.txt) and a file containing the top connected metabolites in the model (MethylocellaMets.txt), as well as the RAST annotation in Excel format (Methylocella.xls), can be downloaded from https://github.com/SergioBordel/ModelsMethanotrophs

### Enzyme activity tests

Isocitrate lyase and malate synthase (experiments on acetate) were assayed following the method of Dixon and Kornberg [26], adapted for a 1 ml volume. For isocitrate lyase, the reaction mixture contained; potassium phosphate buffer pH 7.0 (final concentration 100 mM), MgCl_2_ (6 mM), phenylhydrazine HCl (4 mM) L-Cysteine HCl (12 mM) protein (50—200 µg) and was initiated with DL-isocitrate (trisodium salt) (8 mM). Increase in absorbance at 324 nm, due to the accumulation of glyoxylate phenylhydrazone, was recorded against reactions containing water instead of isocitrate. After a lag of approximately 1–2 min, the reaction was linear for at least 10 min. Rates were calculated using ε_324_ for glyoxylate phenylhydrazone of 1.7 × 10^4^ M^−1^ cm^−1^. Malate synthase was assayed by following the decrease in absorbance at 232 nm due to the breakage of the acetyl-CoA thio-ester bond in the presence of glyoxylate. Reactions (1 ml) contained Tris buffer pH 8.0 (final concentration 90 mM), MgCl_2_ (3.4 mM), acetyl-CoA (sodium salt) (0.05 mM) and protein (100 µg). Absorbance was measured for 5 min before and after addition of glyoxylate (final concentration 0.5 mM). Without substrate, change in absorbance was less than 0.0008 min^−1^. Rates were calculated using ε_232_ for acetyl-CoA of 4.5 × 10^3^ M^−1^ cm^−1^. Alcohol dehydrogenase (experiments on propane) was assayed as previously described [8]. Protein concentration was measured using Bio-Rad Protein Assay reagent, following the manufacturer’s instructions.

### Construction of mutant strains

Unmarked deletion mutants of isocitrate lysase, malate synthase and Msil_1641 were constructed by double homologous recombination, followed by removal of the antibiotic cassette, as previously described [12]. Upstream and downstream regions were generated by PCR, using primers 3157Af / 3157Ar (5′-TCACTGTGCGGCGACTATG-3′ / 5′-TATCGGTACCCGTTGAGGACCGCCTCAAG-3′) and 3157Bf / 3157Br (5′-TATCACGCGTTGCGTCTGCCTTGTTCAGTC-3′ / 5′-TATCGAGCTCCCAGCGCCAGCTGTTCTTC-3′) or 1325Af / 1325Ar (5′-ATCAGTTAACGCCGTGTCGACGCTTATC-3′ / 5′-ATCAGAGCTCCAACCCAGACGCCAAATG-3′) and 1325Bf / 1325Br (5′-ATCAAGATCTGCCAAGCTGGCGTTACCC-3′ / 5′-ATCAGGTACCCGAACGGCTACACGGAAGG-3′), or1641Af / 1641Ar (5′-ATCAGAGCTCAAAGCACGGCCGCTATCG-3′ / 5′-ATCAACGCGTGCGCTTTCGCCCTGATAACC-3′) and 1641Bf / 1641Br (5′-ATCAGGTACCCGTCATTGGGCAACGATAAG-3′ / 5′-GAAACCGCCAATGCATCTC-3′), and cloned into the EcoR1/KpnI and MluI/SacI, or HpaI/SacI and BglII/KpnI, or SacI/MluI and KpnI/EcoRI sites of pCM184 [27], for isocitrate lyase (Msil_3157), malate synthase (Msil_1325) or Msil_1641, respectively.

### Growth of *M. silvestris* on different substrates

*Methylocella silvestris* BL2 and the three marker-free deletion mutants, were grown in 120 ml serum vials, sealed with rubber stoppers, on dilute nitrate mineral salts medium (DNMS) as previously described [12]. Liquid and solid substrates were added to 5 mM, except monomethylamine (10 mM) and methanol and ethanol (0.1% v/v). Gaseous substrates (methane and propane) were added by injection through the rubber stopper to headspace concentrations of 10 or 20% v/v, and their consumption was monitored by gas chromatography (GC) [8]. For cultures on propane, 2-propanol, acetone and acetol were quantified by GC following solvent extraction as previously described [8]

### Measurement of biomass yields

Methane, ethanol and biomass concentrations were monitored over time and the yields were calculating by plotting the produced biomass versus the consumed substrate and obtaining the slope (before reaching limitation by nitrogen). Methane was added to cultures by injection through the rubber stopper to a headspace concentration of 10% v/v, and monitored by gas chromatography (GC) [8]. The initial concentration of ethanol in the medium was 4 mM. Its concentration was quantified by GC, by comparison with standards prepared in water. Culture medium (1 ml) was centrifuged (16,000 × g*, *10 min) and 3 μl of the supernatant injected into an Agilent 7890A gas chromatograph fitted with an HP-PLOT Q column (30 m × 530 μm, Agilent, catalogue number 19095P-QO4). Biomass was calculated from optical density (OD_540_), measured with a Shimadzu UV-1800 spectrophotometer, based on the conversion factor 1 OD ml unit = 0.25 mg dry weight. Methane was measured using a Porapak Q column (Supelco).

## Supplementary information

**Additional file 1: FS1.** List of unique reactions and unique metabolites in the model of M. Sylvestris.

**Additional file 2: FS2.** Gene annotation obtained using RAST.

**Additional file 3: FS3.** Equivalence between gene annotations.

## Data Availability

All the data have been made available at: https://github.com/SergioBordel/ModelsMethanotrophs.
